# The Study of Dental Surgery and the Means Thereto

**Published:** 1882-01

**Authors:** John Tomes

**Affiliations:** England.


### ARTICLE II.

### *The Study of Dental Surgery and the Means Thereto.*

BY JOHN TOMES, F. R. S. ETC., ENGLAND.

Dental surgery has within the present century, by the full consent both of the medical and general public, developed into a well-defined specialty. The medical practitioner refers all dental cases to a dentist where one is at hand, and the general public select him as the fittest to help them in all cases of dental trouble. No apology, therefore, need be offered for the separate practice of dental surgery, neither need arguments be put forward in support of its continuance as a distinct branch of surgical practice. The necessities of society on the one hand and the technical requirements of the dentist on the other hand, have determined the condition of separateness. But this great international meeting affords a fitting occasion to enquire how the accepted condition can for the future be met, so that the public may be best served, for therein rests the sole cause for our presence, either as special or, indeed, as any kind of practitioners whatever.

Utility alone is the excuse for the dentist's existence, and the full recognition of this fact brings us to the question of how and by what available means he can become most useful? How he can best fulfil the trust imposed on him as a specialist, bearing in mind that on account of his supposed superior special knowledge he is consulted, and thus assents to the belief that the dentist is far more capable than the general surgeon in the treatment of dental ailments. Clearly his honor—nay, even his integrity—is pledged to render himself in the highest degree capable of discharging to the fullest the freely-accepted duties. In admitting the social necessity for the presence of the special practitioner, the need for his special education is con ceded, and it is to the wide question of what should be the education of the dental practitioner, for the determination and the development of which, we, as practitioners and teachers, are responsible, that I would call the attention of the meeting.

Before proceeding further, however, let me state that I wish it to be understood that all I have to say upon the subject of dental education applies only to those who have yet to be educated, and to those who possess neither unusual fitness nor unfitness for the pursuit of dental studies. And furthermore, I desire to state that any opinions I may express as to what can and should be done are intended to apply only to education in England. It will be for the representatives of other nationalities to tell us what system of education is most applicable and suitable in their respective countries.

In the first and second decades of the present century dental practitioners were few in number, and for the most part, but not in all cases, members of the medical profession, who at the onset of practice had but a slender knowledge of the duties of the dental surgeon even as they were then understood, or at best they had such an amount of knowledge only as the accident of a good or bad private instructor might impart, in all constructive matters depending from the first upon the assistance of dental mechanists. Other persons commenced their career as young men or boys in the laboratory of a dental practitioner, acquiring therein in the course of an apprenticeship extending over five or even seven years, great manual skill, but whose claim to surgical knowledge at the expiration of pupilage could not be sustained. Yet from this class of persons some of the most distinguished practitioners of the last generation were derived. The one spent those years, when to learn is easy and authority in the teacher is effective, in the acquisition of manual skill; the other in the acquisition of medical, I will not say surgical knowledge, in the strict meaning of the term surgical. Hence it was that practice was approached from two wholly different sides, and resulted in the production of practitioners of two distinct classes; one competent to advise, the other competent to treat, but neither fully competent both to say what should be done and to do it effectively.

Toward the end of the second decade dentists began to increase in number, and each year up to the middle of the century brought new candidates for practice, the vast majority of whom came directly from the dental laboratory, and were, for the most part, inferior in general education to the surgeon, in whose medical knowledge they had no share. Out of this educational difference arose an inter-professional division, not to say jealousy, in which society took but little interest, each person selecting for himself a practitioner from whom he hoped to secure all the advantages that treatment could effect, and the choice as often fell upon the unqualified as upon the surgically qualified practitioner. Among the more intelligent practitioners it came to be freely admitted that dental education, from its one-sided character, was in a very unsatisfactory condition; and after some few years of discussion the opinion was generally accepted that the general and special portion of the dental training should go on simultaneously; so that both manipulative skill and surgical knowledge should be acquired in the days of our youth, when the power to acquire is at its best, and at the only time, indeed, when a high degree of manipulative skill can be acquired.

But we were not the first to recognize the necessity of a systematic dental education. Our American brothers had not only felt but provided for the need in the organization of dental colleges; and we, in following in their footsteps and profiting by their experience, accepted an obligation which should at all times be freely acknowledged.

The history of the organization of the past and present dental colleges of America has been published in "The History of Dental and Oral Science in America," 1876. From this and from Dr. Eliot's address, delivered before the American Academy of Dental Science, 1779, and from the prospectuses of the American dental colleges, I shall take such facts, as should be stated in acknowledgement of the work of our predecessors, and of those differences of method or requirements in education, which differences of attendant social or natural opinions have rendered desirable or necessary.

The distinguished president of Harvard University, Dr. Eliot, in his admirable address on "Dental Education," divided the subjects which constitute the fitting education of the dental surgeon into those who are peculiar to the general and to the special surgeon, and those which are common to both. The general he estimates as three-fifths, and the special subjects as constituting two-fifths of the whole education; and there will be few dissentients to this division. Keeping Dr. Eliot's estimate in mind, but that the medical degree was given and taken on such easy terms it would have been difficult from our standpoint to understand how it was that so many of the dental colleges from the first undertook to educate their students in medicine and surgery, in the presence of schools devoted to these subjects, furnished with all the multitudinous appliances necessary for success in teaching, and with teachers of experience and distinction; and with the further evil of withdrawing the dental student from advantageous association with the general student in the study of subjects common to general and dental surgery. The separation of the students by this limitation to a special school engendered a distinction of social position to the obvious disadvantage of the dental practitioner, whose pretention to the necessary amount of medical or surgical knowledge would be challenged by those who have studied under more favorable circumstances and under the guidance of established teachers. For however little professional education may be forced upon the individual student, there has never been a time when a diligent and determined student could not acquire a competent knowledge of his profession in the medical schools of America or of our own country.

Even a casual study of the organization of the American dental colleges leans to the inevitable impression that the Americans were, and indeed still are, strongly and rightly impressed with the absolute need of thorough training; and although not in a position to enforce the acceptance, yet felt bound to offer to students every inducement to acquire a sound knowledge of the special subjects, and of the requisite manipulative skill. The general subjec s appear to have received less attention, or, at all events, occupied in the college prospectus a less prominent position. In some cases, indeed, it would almost seem that a college faculty thought that a sufficient knowledge of general surgery could be acquired in the study of the special subjects of dental surgery.

It has been urged that dental should come as supplemental to medical knowledge, that the practitioner should be a medical man first and a dentist afterwards. This opinion might be sustained if the position were reversed—a dentist first and doctor afterwards—provided all students, or even the majority, were sufficiently rich in money and time to extend the educational period from four to six years, or from three to four and a half, a condition of things which obtains neither here nor, according to Dr. Eliot, in America.

Four years are alloted to the study of medicine, and the medical student has not an hour to spare for any other subject, hence it becomes needful to determine what subjects in the medical curriculum can be lessened in extent or wholly omitted, so as to find time within the same four years for the effective study of dental surgery as a science and practice. This problem has not, perhaps, been wholly solved, but, as the latest organizers of a complete scheme of compulsory dental education, it is hoped we may claim to have provided the most complete curriculum hitherto brought into a national use. The details of this were determined by a committe of the Medical Council, consisting of the repersentatives thereon of the medical authorities which, under the Dentists' Act, grant dental qualifications, the twenty years' experience of the College of Surgeons of England being placed at their disposal. They reported in favor of, and the Council adopted without material variation, the curriculum originated by the aforesaid College. The unconditional instance upon an attested preliminary education before a person is allowed to commence his professional studies is a feature of great importance in the existing regulations, inasmuch as it insures to the student an amount of knowledge and of mental training which renders him competent to understand without difficulty the language of science and to follow with comparative ease the methods of scientific instruction and investigation. Before this great educational step was taken pupils not uncommonly entered upon their professional studies so poorly informed that much time was lost in the attendance upon lectures which they but very imperfectly understood, and, consequently, at the outset several lectures had to be delivered for the purpose of general instruction, and thus of preparing the student to take advantage of the subsequent course rather than of imparting available medical knowledge. Much has been said against the vast numbers of lectures students have been required to attend, and especially against repetitions, and the objection is no doubt valid now that preliminary education is enforced, and the students thereby enabled to learn as much from one course as they formerly did from two courses. There may be difference of practice, but there can be no difference of opinion here or elsewhere as to the advantage to the student of an attested preliminary education.

The conditions imposed upon dental students are, that he shall subsequent to his having passed the preliminary examination in general knowledge common to the dental and medical student, have devoted four years to the acquirement of professional knowledge; have been engaged during a term of three years in the acquirement of a practical knowledge of clinical dentistry under a competent instructor; have attended and taken part in the dental practice of a recognized dental hospital, or the dental department of a recognized general hospital, during a period of two years; having attended two courses, or not less than twenty-four lectures, on dental anatomy and physiology, human and comparative; two courses, or not less than twenty lectures, on dental surgery; and not less than twelve lectures on metallurgy, and a like course on mechanical dentistry.

These then are the subjects and conditions which take the place of those remitted from the medical curriculum, and who can justly say they do not impose a tax equal to the remission, upon the intelligence, the industry and the time of the student? It may indeed be contended that a greater load is imposed, for it is the opinion of those engaged in instruction, and of those recently instructed, that nothing can be remitted from the special division of the curriculum. The hospital attendance must be exacted almost day by day during the two specified years, in order to attain adequate manipulative skill, without which the practitioner would be as the musician who cannot play, the artist who cannot draw, the sculptor who cannot use the modeling tool or the chisel, or the dental critic who should be able to surpass, but cannot equal, the work he condemns in others. It is one thing to know the scientific principles of the art, but it is quite another to carry them into effect. This requires an amount of manipulative power, which can only be obtained by long and careful practice under a competent instructor. The fingers must become unconsciously obedient to the will; they must follow it automatically, as the fingers of the skilled pianofortist execute the mental reading of the word he is playing, or as the hand of the sculptor produces the form the mind has conceived. Short of this unbidden obedience of hand the performer would be an amateur and his professional life one long apology.

It will be admitted by all that skill of hand can be attained only by long practice, and few will contend that one time is as good as another for the training. Mr. Fawcett has told us that the blind may be taught a bread-winning trade in their youth, but that adults who have lost their sight cannot acquire sufficient skill to secure independence. We know that successful musicians and artists commence their studies in youth, and have given promise of power before they have attained to manhood. If we turn to the artizan class it will be found that he who fails to acquire skill of hand during his apprenticeship seldom attains to excellence afterwards. There is no reasonable ground for doubting that the hand in youth developes anatomically in the direction of its exercise, and acquires thereby a power in that exercise to which the adult hand seldom attains. These facts have an important bearing upon the question of the time at which the dental student should proceed with his practical education, for the skill needed by the dentist is inferior to none of these. The results of professional examinations fully establish the fact, that the medical and dental curriculum cannot be honestly fulfilled in the same four years. Yet it has been said that the practitioner should be a surgeon first and a dentist afterward, or, in other words, the entrance upon the special division of the dental curriculum, should be delayed until the surgical education is completed, thus deferring the manipulative training to a period when the attainment of excellence is difficult, and in its highest degree, perhaps, impossible. To devote the days of our youth to the acquisition of knowledge we do not intend to increase, to the exclusion of the knowledge by the exercise of which we propose to gain our bread, would be, I contend, a great error, and the more so, as the remitted portions of the medical curriculum can, if desired, be taken up when the dental education is completed.

My strong advocacy of special, must not be interpreted an indifference to medical qualifications. I would give every possible encouragement to the attainment of the latter, not, however, as a substitute for, but as a supplement to the dental degree. Educationally, the relations of the membership to the dental license may be regarded in the same light as the relation of the fellowship to the membership are regarded. This view will indeed take effect in certain appointments. In many of our hospitals, although the membership of the College of Surgeons is a full qualification, the governing body require that their surgical officers shall be Fellows of the College. Whenever the Fellowship of his College is required of a candidate—provided the Fellowship implies a higher degree of professional knowledge than the Membership—it may justly be required of the dental candidate for office that he shall possess the Membership in addition to the dental license of his college.

In reviewing the task imposed on the student, it may be asked whether I have not overstated the amount of special training needed to insure the acquisition of the necessary manipulation power. I would answer, No, with all the emphasis I am capable. For I contend that a high degree of skill of hand is absolutely necessary of professional competence—that competence is necessary to self-respect—and the self-respect is necessary to that professional rectitude, without which personal comfort in practice would be imperilled, and status would be but a shallow fiction.

Furthermore, that with the existing opportunities a high degree of skill can be gained by perseverance and due expenditure of time in pupilage, and that it is the bounden duty of the teacher to press, and for an examiner to demand, its possession.

Such, then, are the lines upon which the study of dental surgery have been drawn—so drawn as to secure adequate knowledge and skill in the practitioner—and with the view, therefore, to a certain difference in *kind*, but to equality in *degree*, between the compulsory education of the medical and of the dental practitioner.—*Dental Miscellany.*

